# Adsorption behavior of rhamnolipid modified magnetic Co/Al layered double hydroxide for the removal of cationic and anionic dyes

**DOI:** 10.1038/s41598-022-19056-0

**Published:** 2022-08-26

**Authors:** Asiyeh Kheradmand, Mehrdad Negarestani, Sima Kazemi, Hadi Shayesteh, Shahrzad Javanshir, Hossein Ghiasinejad

**Affiliations:** 1 Department of Civil and Environmental Engineering, Iran University of Science and Technology, Tehran, Iran; 2 Department of Chemistry, Iran University of Science and Technology, Tehran, Iran; 3 School of Chemical, Petroleum and Gas Engineering, Iran University of Science and Technology (IUST), Narmak, Tehran, Iran; 4 Pharmaceutical and Heterocyclic Compounds Research Laboratory, Chemistry Department, Iran University of Science and Technology, Tehran, Iran

**Keywords:** Environmental chemistry, Chemistry, Chemical engineering

## Abstract

In the present research, magnetic rhamnolipid-Co/Al layered double hydroxide (MR-LDH) was synthesized to uptake methylene blue (MB) and reactive orange 16 (RO16) from aqueous solution. The main parameters, including pH, adsorbent dosage, contact time, and initial analyte concentration, were optimized to achieve the best adsorption efficiency. Accordingly, the elimination of MB on MR-LDH is improved in the basic medium due to the electrostatic interactions between the negative charge of MR-LDH and the positive charge of MB dye. In contrast, the acidic medium (pH = 3) was favored for RO16 adsorption because of hydrogen bonding between the protonated form of azo dye and protonated hydroxyl groups at the surface of MR-LDH. The calculated maximum adsorption capacities for MB and RO16 were 54.01 and 53.04 mg/g at 313 K, respectively. The Langmuir model, which assumes monolayer adsorption on the adsorbent surface, provides the best explanation for the adsorption of both dyes (R^2^ = 0.9991 for MB and R^2^ = 0.9969 for RO16). Moreover, the pseudo-second-order kinetic model best described the adsorption process for MB (R^2^ = 0.9970) and RO16 (R^2^ = 0.9941). The proposed adsorbent maintains stable adsorption performance for four consecutive cycles. After each adsorption process, MR-LDH is easily separated by an external magnet. The findings show that MR-LDH was found to be an excellent adsorbent for the removal of both cationic and anionic organic dyes from aqueous solutions.

## Introduction

As a result of the continuous release of pollutants into the environment especially water, the removal of the industrial effluents, including leather, printing, textile, refineries, plastic, and petroleum industries, has become one of the global challenges^1–5^. Due to their slow decomposition and toxicity, dyes can cause irreparable damage to the environment ecosystem and lead to severe problems in aquatic animals and humans^6–8^.

Textile dyes are classified according to their functional groups: nitro, nitroso, azo, anthraquinone, indigo, sulfur, and so on^9,10^. These dyes are recalcitrant, non-biodegradable, bioaccumulative, toxic, and carcinogenic and have harmful effects on the environment, even at low concentrations^11–14^. It is also common practice to categorize dyes according to the charge that is left on their particles after dissolution in an aqueous medium. These categories include anionic (which includes direct, acidic, and reactive dyes), cationic (which includes all basic dyes), and non-ionic (dispersed dyes)^15,16^.

Methylthioninium chloride, commonly called methylene blue, is a water-soluble non-biodegradable cationic dye belonging to the thiazine family with a pka of 3.8. Reactive Orange 16, a highly water-soluble recalcitrant and xenobiotic monoazo dye with a pka of 3.75, is inherently hazardous and has carcinogenic and mutagenic effects in humans^17–20^. Therefore, removing dyes from wastewater is considered an environmental challenge^21,22^.

Diverse techniques have been employed to eliminate synthetic dyes from pollutant water including filtration, flocculation, biological treatment, coagulation, adsorption, extraction, membrane separation, photocatalytic degradation, and oxidation^23–26^. Some of these traditional methods have been restricted due to being complex, time-consuming, and uneconomical^27^. Thus, finding the most efficient and simple dye wastewater treatment method is necessary^28^. In recent decades, adsorption has garnered a lot of attention as a more preferred method due to its flexibility and simplicity in design, as well as its insensitivity to toxic contaminants and its lack of generation of toxic materials^2,25,29–31^. The adsorption efficiency highly depends on the properties of the adsorbent^30^. Tradition adsorbents including clay, biochar, chitosan, zeolite, silica, or synthetic adsorbents including active carbon, polymers, mesoporous carbon material, waste rubber tires or filter membranes have been tested to remove contaminants from wastewater^32–34^.

One of the potent adsorbents is a hydrotalcite-like layered structure known as layered double hydroxide (LDH), bearing an adjustable usage density and significant chemical uniformity^35^. Due to the versatility of structural composition, morphology, and several synthesis strategies, LDH can be easily manipulated for specific adsorbent processes with improved performance^36,37^. Its structure consists of divalent and trivalent metal ions in the layers with guest charge-balancing anions intercalated between the layers^36,37^.

This structure greatly contributes to its anionic exchange capability depending on synthetic conditions and the application goal^38^. The general formula for LDH is given as [M_1-x_^2+^M_x_^3+^(OH)_2_]^x+^[A^n-^]_x/n_·zH_2_O, in which M^2+^ and M^3+^ display divalent cation (e.g. Mg^2+^, Ca^2+^, Zn^2+^, Ni^2+^, Cu^2+^, Co^2+^, and so on.), trivalent cation (e.g. Al^3+^, Fe^3+^, Cr^3+^, Ga^3+^), respectively^39,40^. The common anions that present interlayer of LDHs are sulfate, carbonate, nitrate, hydroxide, chloride, and bigger anion like polyoxometalates^41,42^.

The total charge of LDHs is positive because the anion's negative charge helps hold the positively charged brucite-like sheets through electrostatic attractions^43,44^. Various features of these layered materials like high specific surface area, low cost and facile synthesis, ion-exchange capability, and swelling abilities make them suitable for use as a dye adsorbent^45–47^. There is a significant class of amphiphilic molecules known as surfactants. These molecules have head and tail parts, and they have the ability to attract both polar and non-polar species. They act as a bridge between the air and the liquid as they accumulate on the surface while simultaneously lowering the surface tension of the species they accumulate on^48^. Rhamnolipids (RL), anionic glycolipid biosurfactants, are provided by various bacteria. These materials are a potentially good alternative to synthetic chemical surfactants for pharmaceuticals and cosmetics owing to their advantages, including low toxicity and being environmentally friendly^49^. Rhamnolipids contain 3-(3-hydroxyalkanoyloxy)alkanoate, with hydrophobic acyl chains attached to a hydrophilic moiety which is created by one or two rhamnose molecules^50,51^. Textile dyes, for instance, can benefit from an expanded assessment of biosurfactants' application, especially rhamnolipid-based ones^49^.

Recently, a series of LDH with various metals (Mg, Zn, Mn)-Fe molar ratios were prepared to eliminate dye molecules from wastewater^52,53^. Results showed that the Mg-Fe molar ratio of 3:1 was the optimal efficiency (Mg-Fe LDH: 71.94 mg/g at 298.15 K) for MB removal^3^. In another study, a 3D Mg/Al LDH was synthesized to uptake the anionic Acid Orange 7 (AO7) as well as cationic MB by employing sodium dodecyl sulfate as a modifier. The maximum removal capacities were reported for AO7 and MB as 485.6 and 58.3 mg/g, respectively. Moreover, the regeneration experiments showed that Mg/Al LDH could be recycled five times. Authors also claim that their adsorbent provided a promising platform for removing ionic dyes using 3D-LDHs^54,55^.

Herein, by employing magnetic rhamnolipid and Co/Al LDH, a powerful platform was prepared to assess the removal rate of two organic dyes, i.e., MB and RO16, from effluent wastewater for the first time. The proposal platform, namely MR-LDH displayed the significant capability to remove both dyes from the aqueous solution. Moreover, the magnetic nature of the adsorbent provides a suitable condition for taking the spent adsorbent out from the working solution for repetitive cycles.

## Materials and methods

### Materials and equipments

Rhamnolipid (> 90%) and metal salts (i.e., Fe, Co, and Al salts 99.9%) were provided from Sigma and Aldrich Company, respectively. Other chemicals, including sodium acetate (99.5%), ethylene glycol (99%), MB (99%), RO16 (99%), and formamide (99.9%), were obtained from Merck Company in analytical grade. All materials were employed with no purification.

Different methodologies were used to establish the sample's composition, including powder X-ray diffraction (XRD) with monochromated Cu–Kα radiation (λ = 1.54056 A). A Nicolet 100 Fourier transform infrared (FTIR) spectrometer was used to collect the FT-IR spectroscopic data, which covered the wavelength range of 400–4000 cm^-1^. The shape and particle size of the particles were also determined using transmission electron microscopy (TEM) (TEM Philips EM208S 100 kV). Another tool used to evaluate the morphology of the materials was the scanning electron microscope (SEM) TESCAN VEGAII (Czech Republic). Using a vibrating sample magnetometer, the magnetic characteristics of the samples could be determined. Utilizing an ultraviolet–visible spectrophotometer, the maximum absorbance wavelengths were used to determine the quantities of dyes in the aqueous solutions (DR6000).

### Synthesis of magnetic LDH

3.25 g of FeCl_3_·6H_2_O and 8.64 g of CH_3_COONa·3H_2_O were dissolved in 80 mL of ethylene glycol at 313 °C under solvothermal conditions to produce the MR-LDH. A light brown solution appeared. Then, it was sealed with a polytetrafluoroethylene liner. After that, the reactor containing the as-prepared solution was heated at 473 K within 8 h. The obtained powder was collected followed by washing with distilled water and ethanol several times to eliminate impurities, followed by drying for 9 h at 333 K. The final black powder is magnetic Fe_3_O_4_. Under sonication at 318 K, the urea method was carried out to go on the process. According to this technique, Al(NO_3_)_3_·9H_2_O and Co(NO_3_)_2_·6H_2_O were dissolved in distilled water, resulting in 0.5 M solution. 3 g/L of obtained Fe_3_O_4_ was added to the solution to create MR-LDH. Then, after 10 to 12 min, it was sonicated. A water-ammonia solution (volume ratio 4:1) was added to the suspension to enhance precipitation at pH 9–10. The solution was filtered under vacuum after 20 h at ambient temperature. Then, it was washed four times and dried at 353 K within 24 h to produce the magnetic product powder.

### MR-LDH preparation

**Figure 1:**
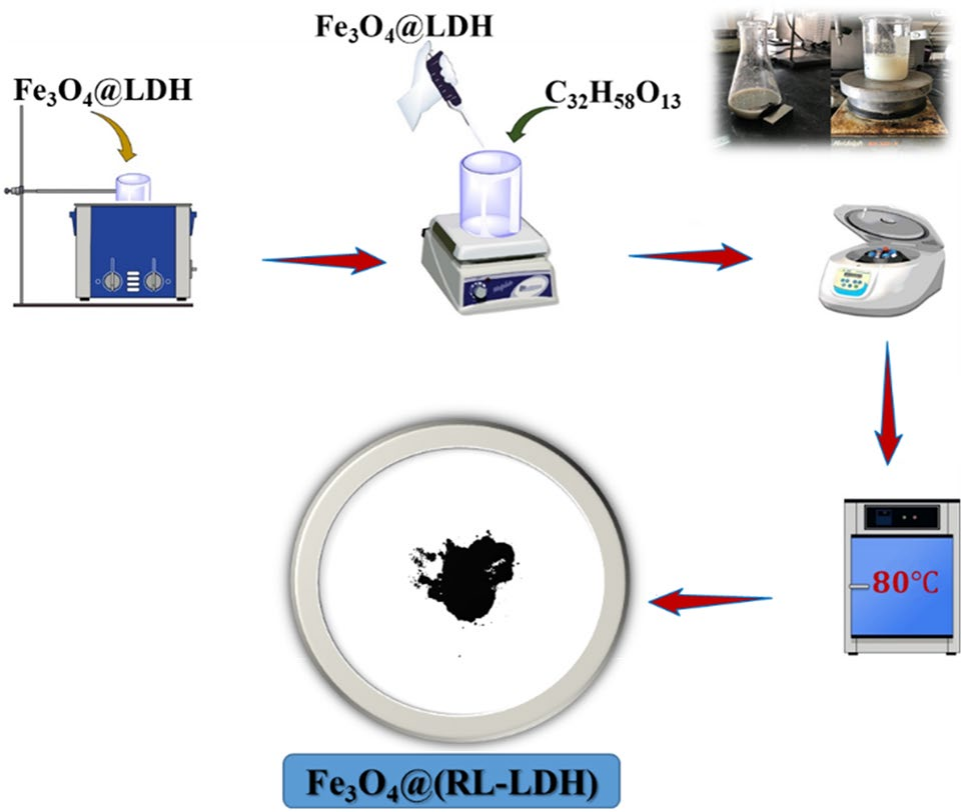
The synthesis process of Fe_3_O_4_@RL-LDH.

For obtaining nanocomposite of MR-LDH, delaminating–reassembling method is employed. Accordingly, 0.4 g of the magnetic core in formamide (25 mL) was stirred, followed by sonicating within 20 min. Then, 20 mL of the suspension was mixed with 20 mL of RL/NaOH 0.1 M aqueous solution containing 1 g of rhamnolipid under weak stirring within 30 min. The suspension was then separated using a magnet and rinsed three times with distilled water/ethanol. Finally, the MR-LDH powder was then produced by drying the product at 353 K. Schematically, the synthesis process of Fe_3_O_4_@RL-LDH illustrates in Fig. 1.

## Results and discussion

### Characterization

**Figure 2:**
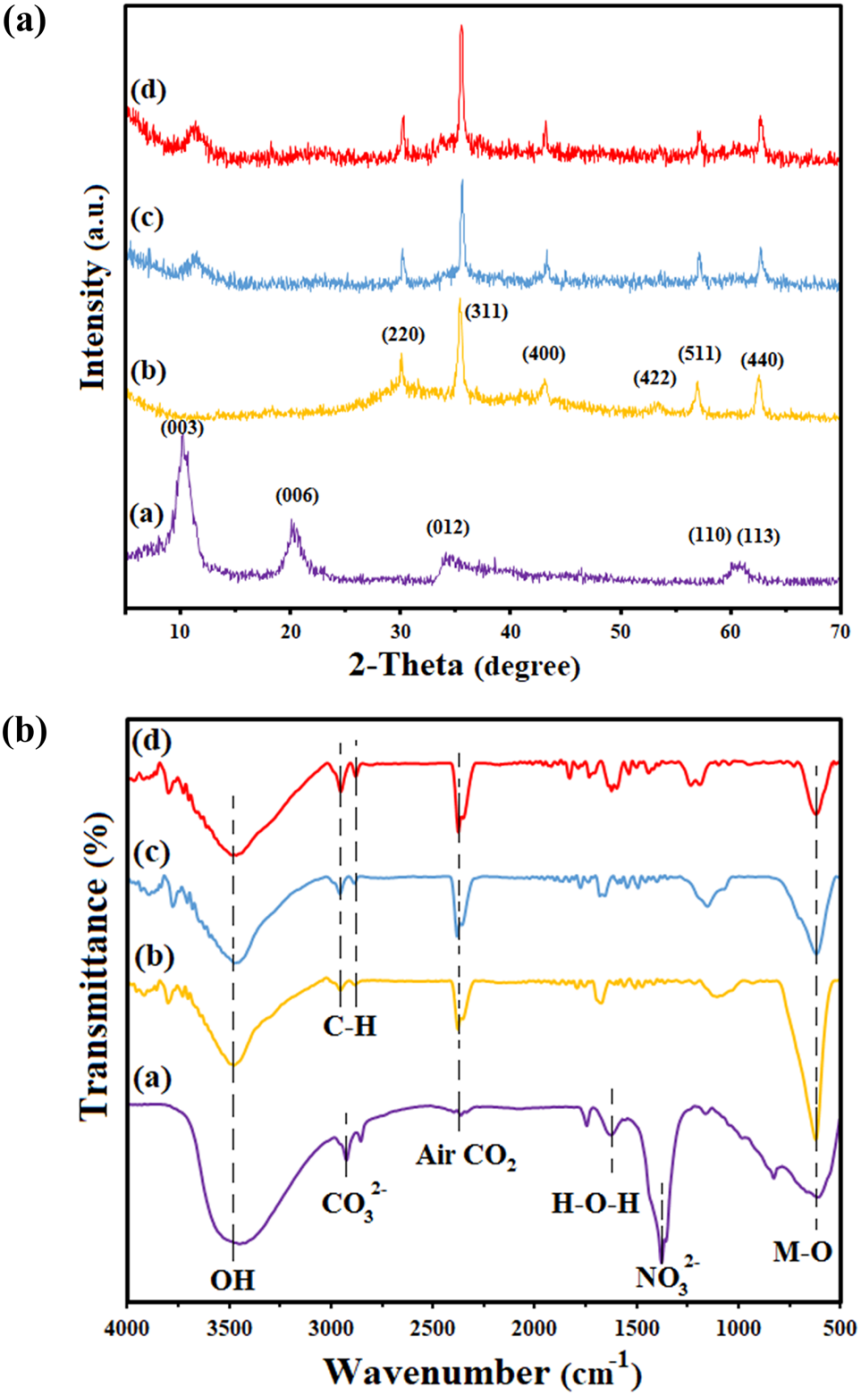
XRD pattern (**a**) and FTIR spectra (**b**); (**a**) LDH, (**b**) Fe_3_O_4_, (**c**) Magnetic Co/Al LDH, and (**d**) MR-Co/Al LDH.

The powder XRD technique was employed to characterize the as-prepared samples. The patterns of all materials are in accordance with simulated pattern (Fig. 2a). The typical peaks of LDH were seen at the diffraction plans (003), (006), (012), (110), and (113). The reflections of (003) and (006) are aroused by hydrotalcite-type material. The strength of the peaks is proportional to the degree of crystallinity of the sample along a certain axis of the sample. Furthermore, the significant peaks in magnetic nanoparticle patterns, i.e., Fe_3_O_4_, at 2θ = 30.2°, 35.5°, 43.3°, 53.6°, 57.5°, and 62.5° are attributed to the reflection planes of (220), (311), (400), (422), (511), and (440), respectively. The distance between the layers of MR-LDH obtained 3.44 nm according to the (003) diffraction peak, which is greater than that of one of the Co/Al-LDH (0.89 nm). This difference is due to the presence of the RL anion within the layers. Furthermore, In XRD patterns of the magnetic core and MR-LDH, characteristic peaks of Fe_3_O_4_ and LDH were also observable.

FTIR spectra of the materials are demonstrated in Fig. 2b. The peaks at 800 cm^-1^ were attributed to the stretching vibrations links between metal and oxygen (M–O) in the samples. Surface OH of Co/Al LDH layers and hydroxyl stretching vibration of coordinated water were observable at 3500 cm^-1^ in all materials' broadband spectra. The peak of LDH at 2900 cm^-1^ explains the interaction of interlayer CO_3_^2^ and water molecules. The stretching vibration of LDH was discovered to be the interlayer nitrate anion at 1385 cm^-1^. The vibration of the Fe–O bond is depicted in the pattern of Fe_3_O_4_ as a peak at 582 cm^-1^, but it is diminished in the composites' spectra. The vibration mode of the nitrate anion in MR-LDH disappeared, but the C-H stretching vibration caused the new bands at 2928 and 2856 cm^−1^.

**Figure 3:**
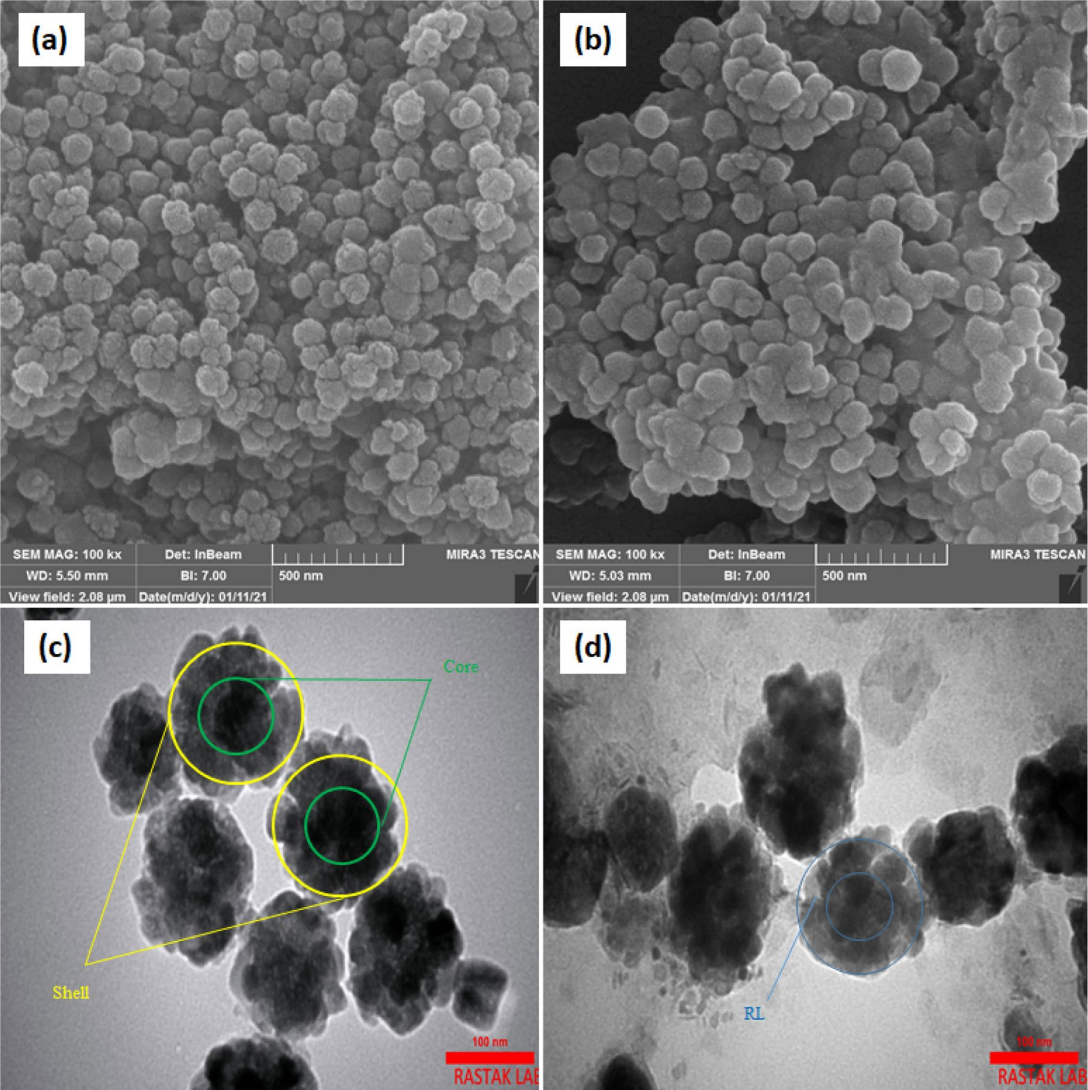
SEM and TEM images of magnetic LDH (**a**), (**c**) and MR-LDH (**b**), (**d**).

SEM and TEM pictures of the magnetic core and as-prepared MR-LDH are shown in Fig. 3. As illustrated, LDH before modification with RL has a lamellar structure and remains in this shape in the presence of RL. According to the TEM images, the Fe_3_O_4_ core is encapsulated by LDH nanosheets resulting in a core–shell composite structure. The composite's average size was calculated to be around 100 nm. Furthermore, Fig. 3 proves that RL was placed between the layers of LDH, leading to aggregating the final composite.

**Figure 4:**
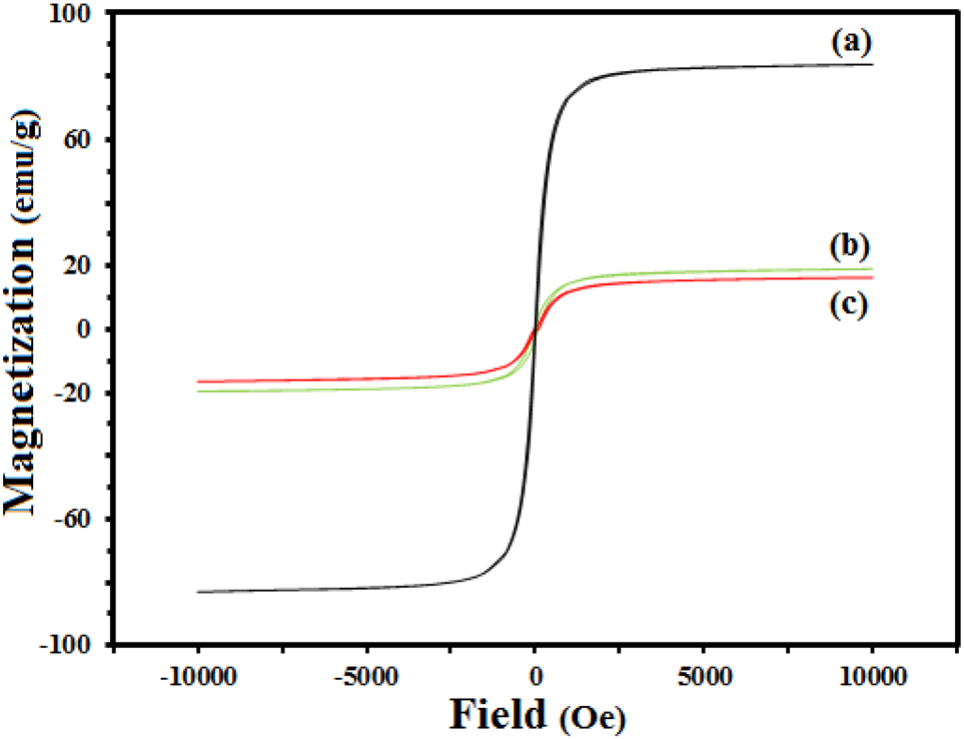
Magnetization curves of (**a**) Fe_3_O_4_, (**b**) magnetic LDH, and (**c**) MR-LDH.

Figure 4 provides the magnetization curves of all the samples. An acceptable super-paramagnetic property is observable, according to the plots. Compared to magnetic Fe_3_O_4_, the saturation magnetization of the core and MR-LDH declines because of the addition of nonmagnetic moieties (i.e., LDH sheets). The samples' hysteresis and coercivity have faded away. Furthermore, because of the presence of RL within the layer, the amount of saturation magnetization of MR-LDH is significantly lower than the value of magnetic LDH.

### Adsorption experiments

#### Effect of solution pH

**Figure 5:**
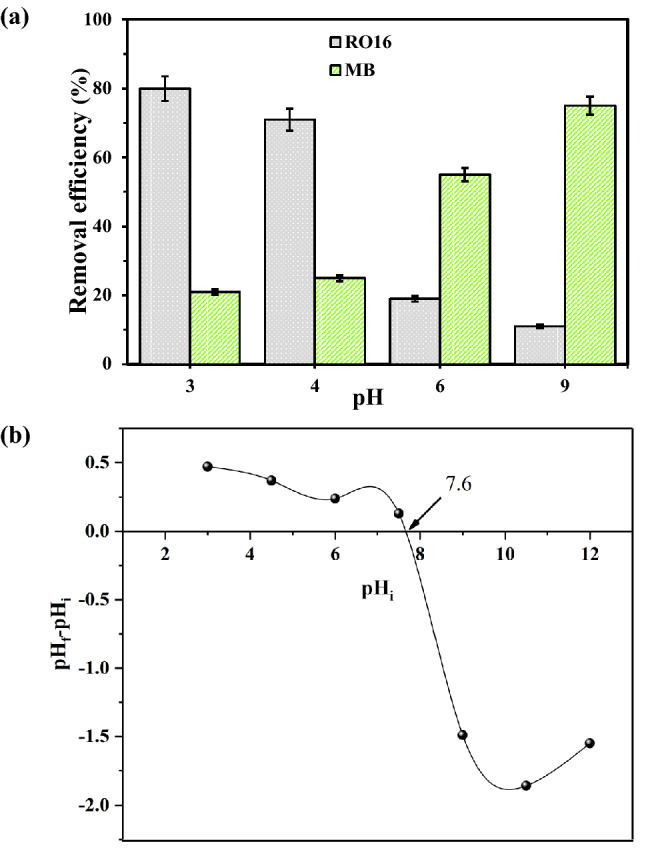
(**a**) The effect of pH of the solution on the MB and RO16 adsorption and (**b**) determination of pH_pzc_.

The surface charge of the adsorbent and adsorbate is highly dependent on the pH of the solution. This implies that the deprotonation (or protonation) of a dye must be considered^30^. The pH can change the ionization degree, the adsorbent surface charge, and the molecular structures of the adsorbates. So, the solution pH determines the kind of interaction between the adsorbent and dyes through the ionization of species in the solution. As depicted in Fig. 5a, with decreasing pH to the acidic range, the adsorption efficiency of MB declines. Based on the plot, the optimum pH for MB removal was found to be 9, which will be used for further experiments. Considering the MB structure, it bears π-conjugated electron density, but the proposal adsorbent does not contain π-conjugated electrons leading to ruling out π-π stacking interaction. While positively charged adsorbent may interact with MB as cation-π interaction effectively in the upper and bottom of the MB plane. Moreover, hydrogen bonding can form between hydroxides of the adsorbent and the nitrogen site of MB, leading to effective adsorption. But, the scenario is completely different for RO16 adsorption. It does not bear an expanded π-conjugated structure. As illustrated in Fig. 5a, adsorption efficiency reached the highest value when pH was reduced to 3. At low pH, this dye ionizes into anion form, affecting electrostatic interaction with the positively charged adsorbent. Furthermore, the existence of hydroxyl groups in the RO16 structure results in hydrogen bonding with OH sites of the proposal adsorbent. As a result, pH 3 was chosen as the best value for subsequent studies aimed at RO16 removal. Furthermore, the point of zero charges (pH_pzc_) of the adsorbent was obtained to be equal to 7.6 (Fig. 5b). The proper interaction behavior of MR-LDH for MB and RO16 adsorption can be described using pH_pzc_. MR-LDH is negatively and positively charged at pH > pH_pzc_ and pH < pH_pzc_, respectively. Thus, at pH > pH_pzc_ the electrostatic interaction between adsorbent and analyte is repulsion and attraction for RO16 and MB, respectively. On the other hand, at pH < pH_pzc_, it happens completely the opposite way.

#### Effect of MR-LDH nanocomposite dosage

**Figure 6:**
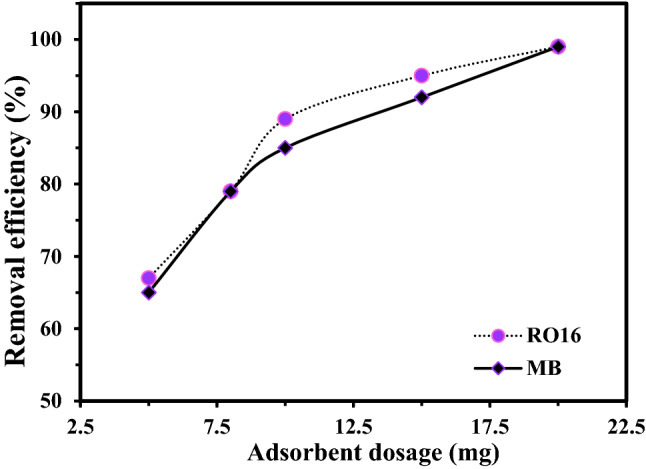
The effect of adsorbent dosage on the MB and RO16 removal.

The adsorbent dosage also affects the adsorption behavior. Generally, increasing the adsorbent dosage leads to increasing the adsorption because more active sites will be accessible for the analyte. In contrast, higher dosages may cause adsorbent agglomeration and lose the active sorption sites. In other words, the adsorbent seeds stack to each other and do not let the analyte interact with the adsorption sites. Therefore, finding the optimized adsorbent dosage is one of the major stages in contaminant removal until they can perform separately. Due to the adsorbent's unsaturated active sites, the adsorption capacity is increased and accelerated. The plots of the removal efficiency of MB and RO16 at different adsorbent dosages are drawn in Fig. 6. According to the findings, MB removal efficiency increased as adsorbent dosage increased up to 15 mg of MR-LDH. Then, the process reaches equilibrium. In the case of RO16 adsorption, 10 mg was the optimal adsorbent dosage. After the optimum values, adding more adsorbents leads to agglomeration of the active sites on the adsorbent, decreasing the adsorption efficiency^56^. Therefore, adjusting the adsorbent dosage to optimum value is also beneficial from the economic aspect.

#### Effect of dye concentrations

**Figure 7:**
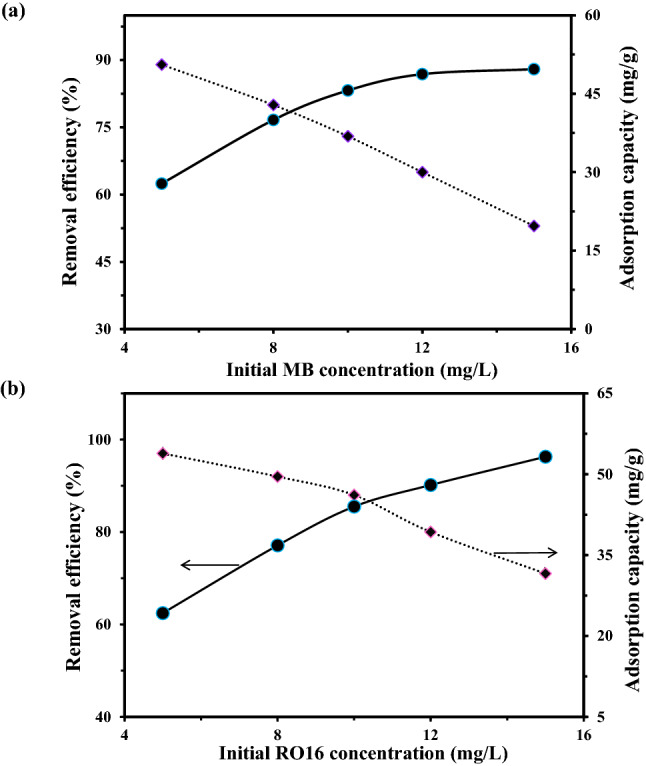
The effect of initial dye concentration on the (**a**) MB and (**b**) RO16 removal.

Another aspect that needs to be taken into consideration is the effect of the initial dye concentration. Notably, an increase in dye concentration has perceptible effects up to a critical limit. Additionally, the adsorbent tends to adsorb high concentrations of dye molecules that are proportional to the accessible sorption sites on the adsorbent's surface. As the surface saturation is occurred, remarkable dye adsorption will be observed. Thus, the adsorption of MR-LDH toward MB under various dye concentrations (5, 8, 10, 12, and 15 mg/L) are displayed in Fig. 7a. Accordingly, as the concentration of MB rises from 8 to 15 mg/L, the ability of MR-LDH to bind to it decreases. 8 mg/L was the best initial concentration for MB uptake (Fig. 7b). In the case of RO16 removal, the highest adsorption efficiency was observed in 10 mg/L initial concentration. After optimum values, the active sites of the adsorbent were saturated, leading to decreasing the adsorption efficiency. Because the vacant space between the layer of MR-LDH is limited. By replacement of interlayer anions of the adsorbent with contaminants, the adsorption is performed. Therefore, this space can accept the optimum amount of analyte, leading to effective adsorption. In this regard, after saturation of active sites of the interlayer of MR-LDH, further contaminants cannot diffuse onto the adsorbent.

#### Effect of contact time

**Figure 8:**
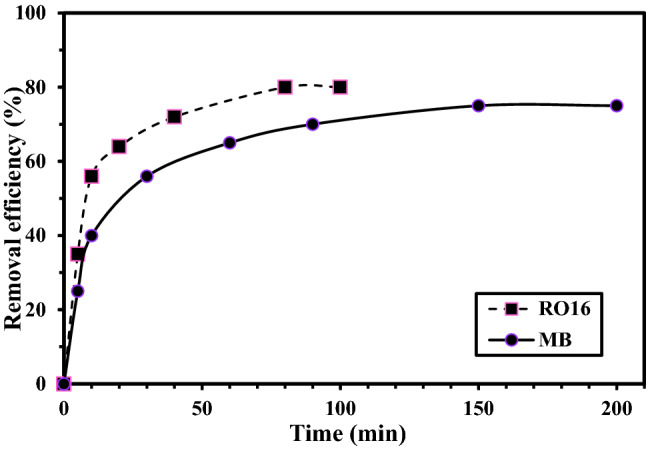
The effect of contact time on the MB and RO16 removal.

Increasing contact time can negatively and/or positively affect the adsorptive removal of dyes. During the upward part of the plot, the contact of dyes and adsorbent leads to effective adsorption because the active sites have not been saturated yet. When the equilibrium between the sorption sites and dye molecules is established, additional reaction time does not affect adsorption because the sorption sites become saturated during contact time, and no more unoccupied space is available for additional dye adsorption. The optimum contact time for dye removal is shown in Fig. 8; in this case, MR-LDH was able to remove 75% of the initial concentration of MB after only 150 min. Then, the adsorption processes remained unchanged within passing time illustrating the active sites of MR-LDH were saturated. In a report, magnetic-based polymers Core@double-shell structured magnetic halloysite nanotube was eliminated MB during 3 h^57^. For RO16, in less than 40 min, about 97% of the analyte was taken up and no more dye remained in the solution. But in previous literature, the time of 50 min was reported for removal of RO16 by chitosan-fly ash/Fe3O4^32^.

#### The effect of temperature

**Figure 9:**
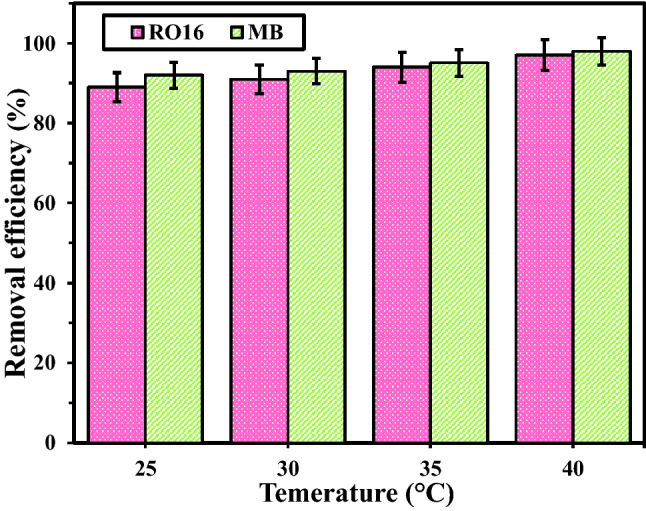
The effect of temperature on the MB and RO16 removal.

In Fig. 9, the optimization of temperature was carried out. As can be seen, for both dyes, 40 ºC has the best result and leads to the highest adsorption efficiency. Since with increasing the temperature, the adsorption is elevated, it can be said that both reactions are endothermic.

### Adsorption isotherms

To analyze experimental data from the adsorption process, the distribution of the dyes among the aqueous solution and MR-LDH at the adjusted temperature and discuss the equilibrium of adsorption, some well-known adsorption isotherm equations, including Freundlich, Langmuir, and Temkin will be introduced here^58^. Firstly, the non-linear form of the Langmuir isotherm is presented as Eq. (1):

1 $$q_{e} = \frac{{q_{m} K_{L} C_{e} }}{{1 + K_{L} C_{e} }}$$

where q_e_ (mg/g) represents the equilibrium adsorption capacity and q_m_ (mg/g) is the maximum adsorption capacity. K_L_ (L/mg) denotes the model's constant. With Eq. (2), the Langmuir isotherm's basic properties are introduced as a dimensionless equilibrium parameter (R_L_):

2 $${\text{R}}_{{\text{L}}} = { }\frac{1}{{1 + {\text{K}}_{{\text{L}}} {\text{C}}_{0} }}$$

where C_0_ (mg/L) shows the highest initial concentration of the analyte, and K_L_ is the Langmuir constant. R_L_ demonstrates the type of adsorption to be either unfavorable (R_L_ > 1), favorable (0 < R_L_ < 1), linear (R_L_ = 1) or irreversible (R_L_ = 0). This isotherm assumes homogeneous adsorption and single-layer coverage of the MR-LDH surface by dye without any interaction between analyte molecules.

An empirical model for surface heterogeneity and an exponential distribution of the energy and sorption sites of the adsorbent is the Freundlich isotherm. The Freundlich isotherm as a reversible adsorption model is not limited to monolayer formation, which is shown in Eq. (3):

3 $$q_{e} = K_{F} C_{e}^{1/n}$$

where K_F_ (mg/g)/(mg/L)^n^ and n are described Freundlich constant. The value of n illustrated that the adsorption is favorable and placed in the range of 1 to 10.

The adsorption heat between dyes and MR-LDH is determined by the Temkin isotherm. Temkin isotherm assumes interaction between dyes and MR-LDH which is presented as Eq. (4):

4 $$q_{e} = \left( {\frac{RT}{{b_{T} }}} \right){\text{ln}}\left( {K_{T} C_{e} } \right)$$

where R is the gas's universal constant (i.e., 8.314 J/mol. K), T (K) is the temperature, b_T_ (J/mol) points to the adsorption heat constant of Temkin and K_T_ is the Temkin isotherm equilibrium binding constant (L/g).

**Figure 10:**
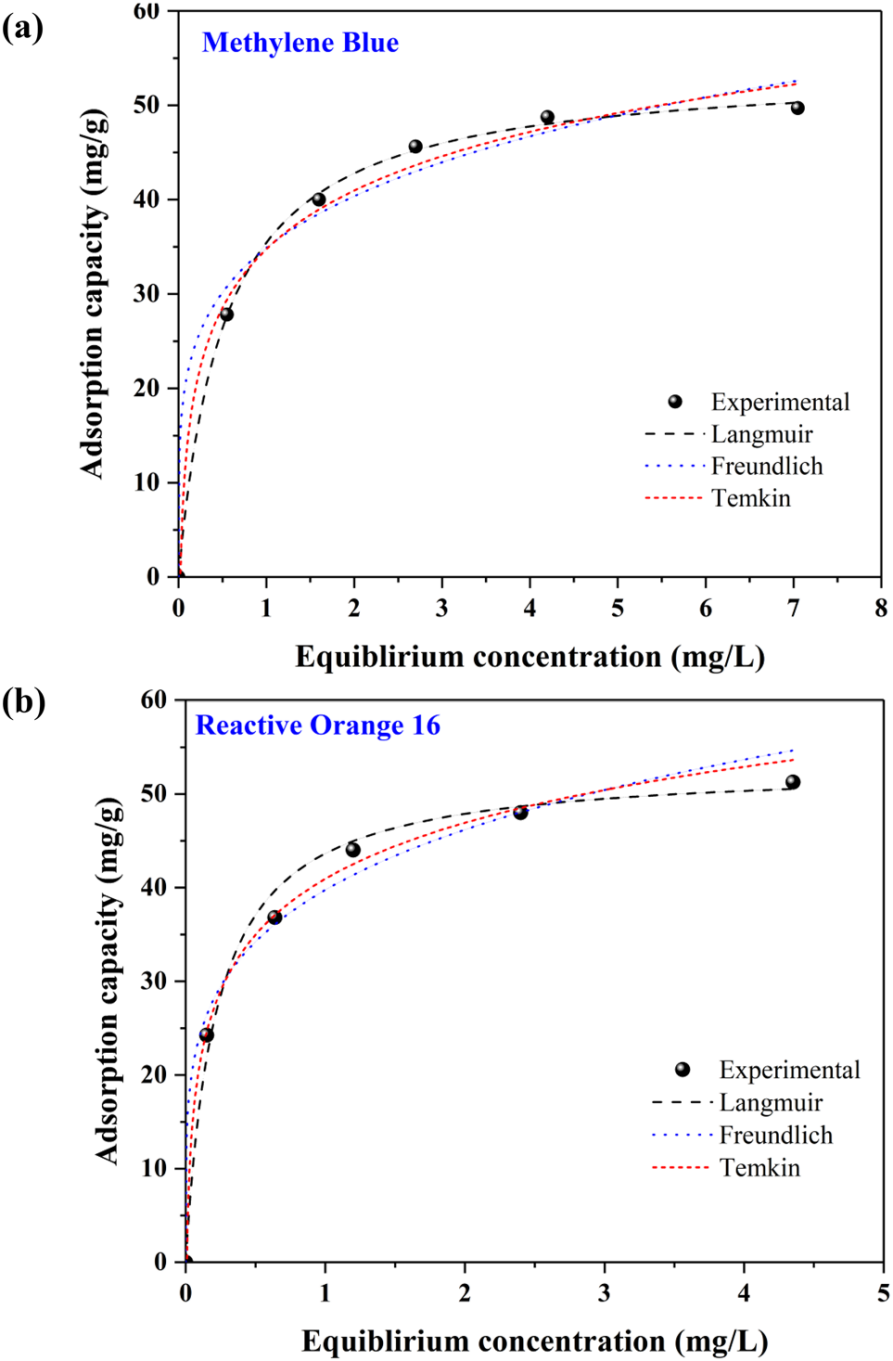
Isotherm fitted curves (non-linear) of (**a**) MB and (**b**) RO16 adsorption.

The adsorption isotherm of MB and RO16 for MR-LDH was assessed at 298 K and the data were analyzed by Freundlich, Langmuir, and Temkin models, respectively. Figure 10 demonstrates the adsorption capacity as a function of the equilibrium RO16 and MB concentration using a non-linear fitting method. In the case of MB removal, the adsorption process followed the Langmuir model, indicating that the adsorption is monolayer and takes place on the particular homogenous sites of the MR-LDH surface. The correlation coefficients of the Langmuir isotherm model were calculated equal to 0.9991, which is significantly higher than the R^2^ values for Freundlich (0.9841) and Temkin (0.9915) models. The Langmuir model has been found to be the best fitting model for describing the adsorption pathway in some previous studies for MB removal. The removal of MB by AC made from coconut leaves with an adsorption capacity of 149.3 mg/g, AC made from lemon peels/sodium alginate with an adsorption capacity of 841.37 mg/g, magnetite/MWCNTs with an adsorption capacity of 55 mg/g, and so on, can be noted among them. In the case of RO16 adsorption, the corresponding plot displays that the adsorption follows the Langmuir model either^59–61^. Therefore, the adsorption of RO16 onto MR-LDH was also monolayer uniform adsorption and no more interaction between the dye molecules was not formed^62^. The correlation coefficient of the Langmuir model for RO16 adsorption was calculated as 0.9969, further proving the fitting model. The correlation coefficients of two other models, including Freundlich (0.9827) and Temkin (0.9885), are relatively lower than the Langmuir value. In Table 1, all numerical investigations of all adsorption models are observable.

**Table 1:** Adsorption isotherm constants for RO16 and MB adsorption on the MR-LDH.

Isotherm models	Important parameters	RO16	MB
Langmuir	q_max_ (mg/g)	53.04	54.01
	K_L_ (L/mg)	4.65	1.91
	R^2^	0.9969	0.9991
Freundlich	K_F_ (mg/g)/(mg/L)^n^	39.76	34.89
	n	4.62	4.75
	R^2^	0.9827	0.9841
Temkin	b_T_	287.02	276.36
	K_T_ (L/g)	114.77	48.23
	R^2^	0.9885	0.9915

### Adsorption kinetics

Kinetic parameters are mainly considered to define the adsorption efficiency because fast kinetics is of great significance in aqueous phase adsorption. To evaluate the kinetic parameters of MR-LDH, the collected experimental results were fitted with four common kinetic models, including pseudo-first-order, pseudo-second-order, Elovich, and Fractional kinetic models. The pseudo-first-order kinetic model is presented in Eq. (5):

5 $$q_{t} = q_{e} \left( {1 - e^{{ - k_{1} t}} } \right)$$

After the integration of the previous equation, the pseudo-first-order is illustrated in the linear form (Eq. 6):

6 $$\ln \left( {q_{e} - q_{t} } \right) = lnq_{e} - k_{1} t$$

The sorption kinetics also was described by the pseudo-second-order model, which is presented by Eq. 7:

7 $$q_{t} = \frac{{k_{2} q_{e}^{2} t}}{{1 + k_{2} q_{e} t}}$$

Following the integration of the above equation, the pseudo-second-order model is denoted by Eq. 8:

8 $$\frac{t}{{q_{t} }} = \frac{1}{{k_{2} q_{e}^{2} }} + \frac{t}{{q_{e} }}$$

where k_1_ and k_2_ are the rates constant of the pseudo-first-order model (min^−1^) and pseudo-second-order model (g/mg.min), respectively.

The third one is Elovich as follows [Eq. (9)]:

9 $$q_{t} = \frac{1}{\beta }ln\left( {\alpha \beta t + 1} \right)$$

The non-linear form of the fractional power equation is as follows [Eq. (10)]:

10 $$q_{t} = k_{p} t^{{v_{p} }}$$

where the antilog of intercept leads to the value of constant k_p_. v_p_ is a constant which is commonly less than unity if adsorption kinetic data fit well into the power function model. q_t_ is the quantity of analyte adsorbed at time t^63^.

**Figure 11:**
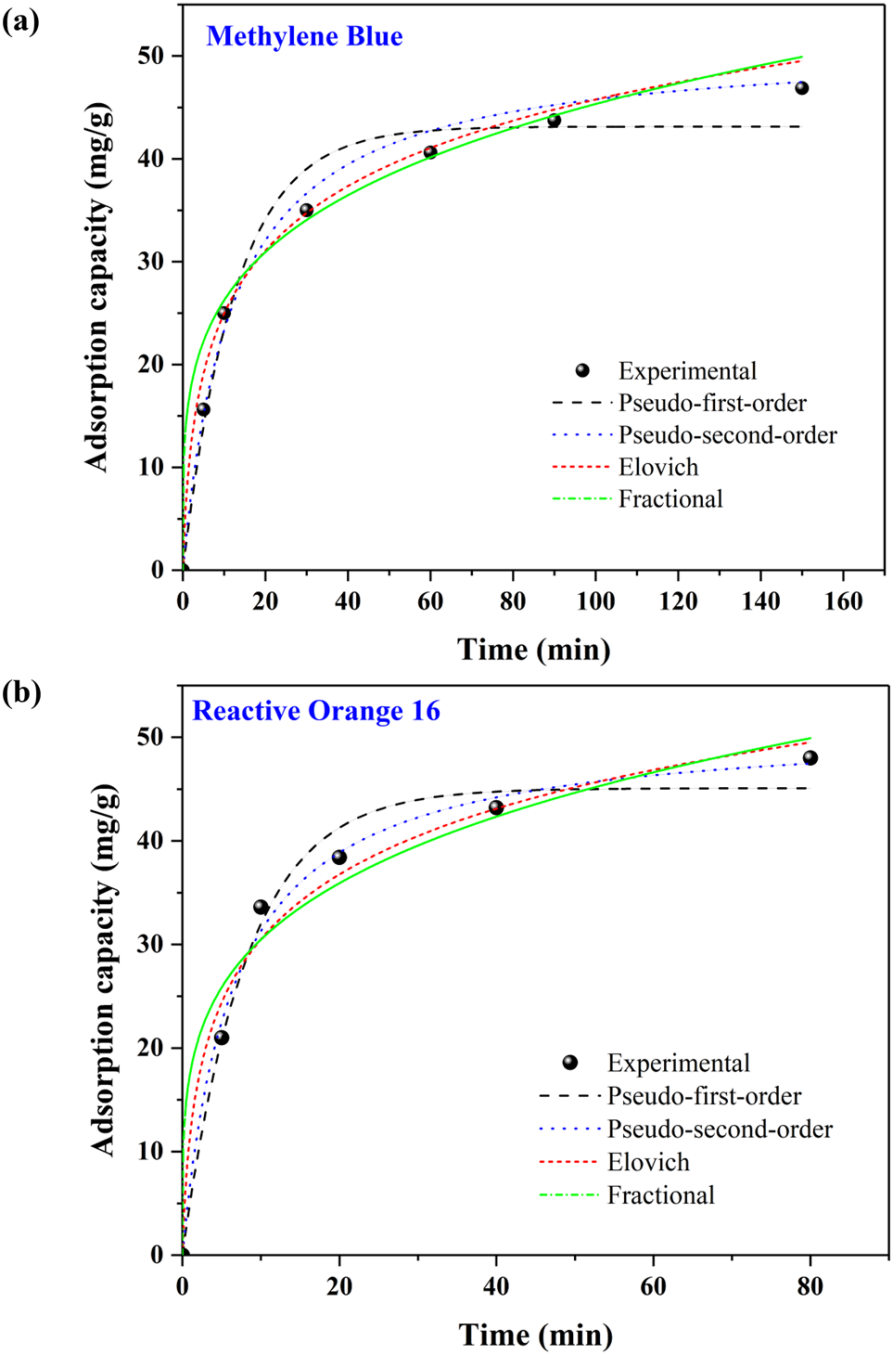
Kinetic fitted curves (non-linear) of (**a**) MB and (**b**) RO16 adsorption.

The plots (non-linear curve fitting) and results obtained from the mentioned models are provided in Fig. 11 and Table 2 for both dyes, respectively. In the case of MB removal, by comparing the correlation coefficients, the pseudo-second-order (R^2^ = 0.9970) was superior to those of the others, including pseudo-first-order R^2^ of 0.9769, and Elovich of 0.9931 and fractional of 0.9780. In addition, the calculated equilibrium capacity value (q_e,cal_ = 48.86 mg/g) using the pseudo-second-order was closer to the corresponding experimental one (q_e,exp_) compared to those of the other models. Thus, the adsorption of MB dye by MR-LDH may be well described by a pseudo-second-order kinetic model. Consequently, electrons are shared or exchanged between the MR-LDH and the positively charged MB dye taken place during chemisorption. In recent research, egg shells derived from industrial waste were utilized to adsorb MB, demonstrating an adsorption capacity of 94.9 mg/g, which was consistent with the pseudo-second-order kinetic model^64^. In another study, finger-citron-residue-based activated carbon removed MB with a capacity of 581.40 mg/g, also fitting with the pseudo-second-order kinetic model^65^. In the case of RO16 adsorption, identical outcomes were observed again. As shown in Table 2, the value of R^2^ for the pseudo-second-order (R^2^ = 0.9941) is closer to 1.0 than that of the pseudo-first-order, demonstrating the applicability of the pseudo-second-order model to describe the rate of adsorption, which assumes that chemisorption may be the rate-limiting step. In a different study that has already been published, a cross-linked composite of natural and synthetic clays with a capacity of 190.97 mg/g for RO16 adsorption was produced^66^. Moreover, another adsorbent for RO16 adsorption with a capacity of 66.9 mg/g was chitosan-fly ash/Fe_3_O_4_, which was fitted with the pseudo-second-order kinetic model^32^. Authors claimed that electrostatic attraction, H-bonding, and π–π stacking interactions are responsible for the adsorption process as the subset of the chemisorption category.

**Table 2:** Kinetic parameters for RO16 and MB adsorption on the MR-LDH.

Kinetic model	parameters	RO16	MB
Pseudo-first-order	q_e,cal_ (mg/g)	45.07	43.14
	k_1_ (1/min)	0.1237	0.0784
	R^2^	0.9859	0.9769
Pseudo-second-order	q_e,cal_ (mg/g)	54.25	48.86
	k_2_ (g/mg × min)	0.0031	0.0019
	R^2^	0.9941	0.9970
Elovich	α	23.82	10.80
	β	0.1076	0.1066
	R^2^	0.9832	0.9931
Fractional	k_p_	17.6385	12.9692
	v_p_	0.2374	0.2672
	R^2^	0.9719	0.9780

### Adsorption mechanism

**Figure 12:**
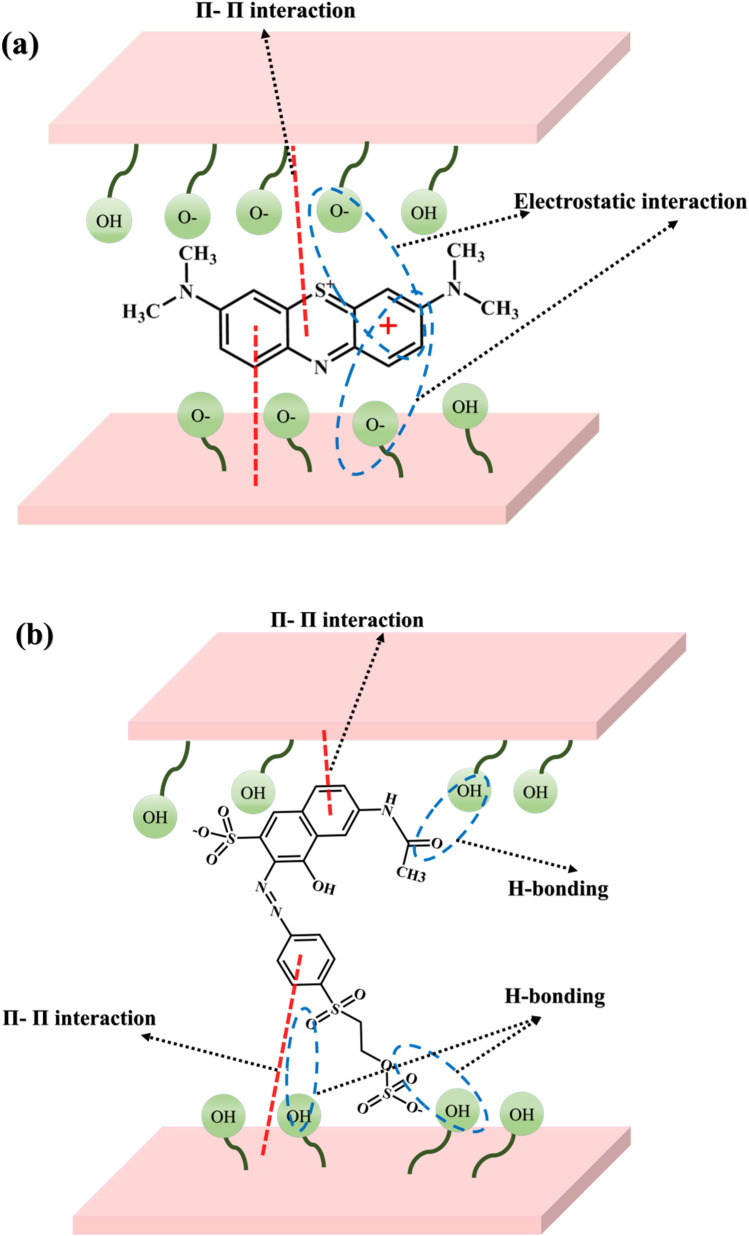
The removal mechanism of (**a**) MB and (**b**) RO16 by MR-LDH nanocomposite.

In general, hydrogen bonds, π–π stacking, electrostatic attraction, base-acid interactions, van der Waals forces, and hydrophobic contact are the main mechanisms involved in the adsorption of water contaminants on adsorbents. Replacement of analytes, and dyes in this case, with the interlayer anion of MR-LDH is the main driving force of the adsorption route. In this approach, various interactions facilitate the adsorption phenomenon. In the case of MB removal, the π-conjugated electrons can interact with positively charged MR-LDH as π-cation interaction. Lacking the π-conjugation plane in MR-LDH rules out the π-π stacking interaction between them. As the adsorption took place at basic pH, partially deprotonation of the LDH can conduct electrostatic interaction with MB, which accelerates the adsorption process. In the acidic pH of adsorption for the RO16 removal, RO16 was partially protonated, which rules out the electrostatic attraction with the adsorbent. In this case, the presence of π electrons density can form π-cation interaction between RO16 and MR-LDH. Moreover, the presence of OH groups can perform hydrogen bonding with hydroxides of MR-LDH. Schematic illustrations of adsorption are provided in Fig. 12.

In addition to the explanations provided, the possibility of diffusion from the solution containing adsorbate to the solid surface can be checked by the Weber-Morris intra-particle diffusion equation. Basically, the intra-particle diffusion equation is used to investigate the controlling step of the adsorption process. The linear form of the intra-particle diffusion equation is expressed as Eq. (11)^67^:

11 $$q_{t} = k_{id} t^{1/2} + {\text{I}}$$

**Figure 13:**
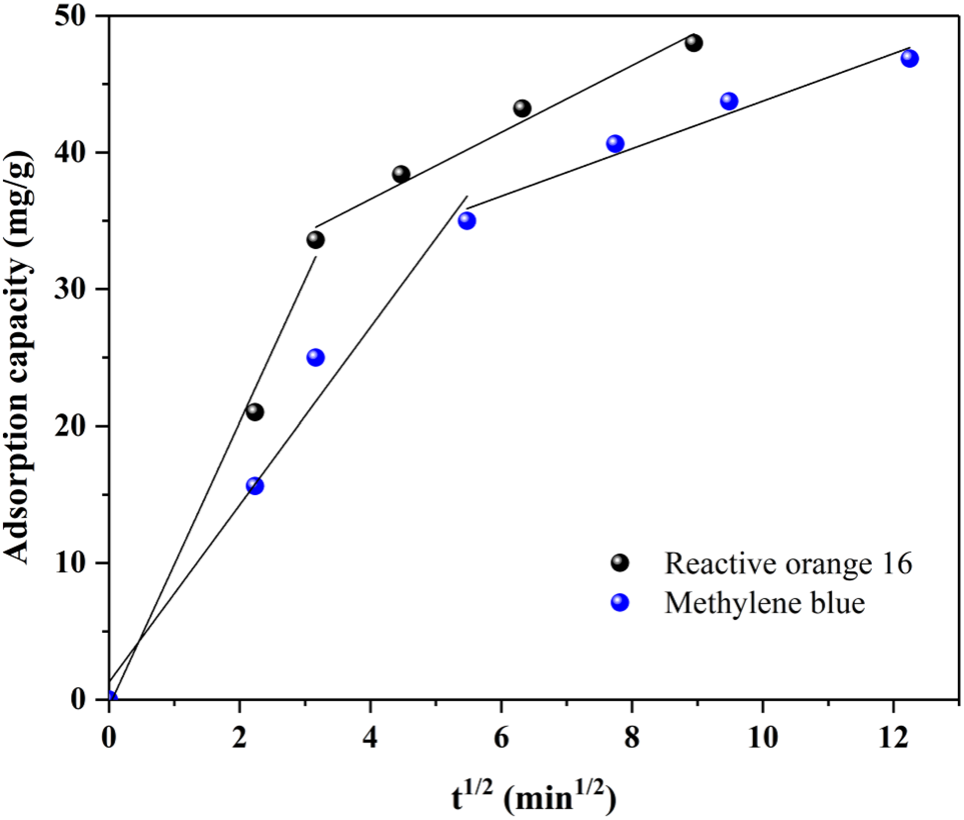
Intra-particle diffusion for removing MB and RO16 onto the MR-LDH nanocomposite.

In the mentioned equation, k_id_ (mg/g min^1/2^) and I (mg/g) represent the intra-particle diffusion rate constant and a constant related to the boundary layer thickness, respectively. Figure 13 shows the changes in adsorption capacity vs. t^0.5^. As it is known, in general, the changes in adsorption capacity with t^0.5^ are not linear, which indicates that the adsorption process is controlled in different stages. The adsorption of MB and RO16 by MR-LDH was conducted in two stages: initial bulk diffusion, followed by intra-particle diffusion into the pores. The changes in slope for both dyes (from high to low) indicate that the external diffusion was faster and that intra-particle diffusion was the controlling phase^68,69^. In Table 3, the values of k_id_ and I as well as the correlation coefficient (R^2^) are given as separate steps in the adsorption process.

**Table 3:** Intra-particle diffusion constants to remove MB and RO16 onto the MR-LDH nanocomposite.

	First adsorption stage			Second adsorption stage		
	$k_{1d}$(mg g^-1^ min^-1/2^)	$I_{1}$	$R_{1}^{2}$	$k_{2d}$(mg g^-1^ min^-1/2^)	$I_{2}$	$R_{2}^{2}$
MB	6.4860	1.2715	0.9771	1.7397	26.3590	0.9633
RO16	10.3970	0.5095	0.9917	2.4430	26.8120	0.9771

### Adsorption thermodynamic

Thermodynamic parameters, including ΔGº, ΔSº, and ΔHº, are also calculated using the following equations [Eqs. (12)–(14)]:

12 $$\Delta {\text{H}}^\circ - {\text{T}}\Delta {\text{S}}^\circ = - {\text{RT}}\ln {\text{K}}_{{\text{D}}}$$

13 $$\Delta {\text{G}}^\circ = \Delta {\text{H}}^\circ - {\text{T}}\Delta {\text{S}}^\circ$$

14 $${\Delta G}^\circ = - {\text{RTlnK}}_{{\text{D }}}$$

where K_D_ points to the distribution coefficient of the adsorption, R and T are the universal gas constant [8.314 J/(mol·K)] and the temperature (K), respectively.

To examine the thermodynamic behavior of MR-LDH toward MB and RO16 adsorption process, the adsorption process was carried out at diverse temperature values. Using mentioned thermodynamic equations and plotting ln(K_D_) against 1/T, ΔHº (kJ/mol) was obtained from the slope of the linear graph, and ΔSº (kJ/mol.K) was calculated using the intercept. All numerical data are gathered in Table 4. As can be concluded in the case of MB adsorption, increasing the temperature increases the removal efficiency (98% at 313 K). Therefore, adsorption is an endothermic process. For RO16 adsorption, increasing the temperature is also favorable for the adsorption process and the highest removal efficiency (97%) was obtained at 313 K. This process is endothermic. Notably, the negative values of ΔG° for MB and RO16 adsorption on the MR-Co/Al LDH at varied temperatures demonstrated their spontaneity.

**Table 4:** Thermodynamic parameters for RO16 and MB adsorption onto the MR-Co/Al LDH.

	$\Delta H^\circ$ (J/mol)	$\Delta S^\circ$ (J/mol K)	$\Delta G^\circ$(J/mol)			
			298 K	303 K	308 K	313 K
RO16	70,984.9	325.343	− 25,967.23	− 27,593.945	− 29,220.66	− 30,847.375
MB	78,625.5	349.287	− 25,462.028	− 27,208.463	− 28,954.898	− 30,701.333

### MR-LDH nanocomposite reusability

**Figure 14:**
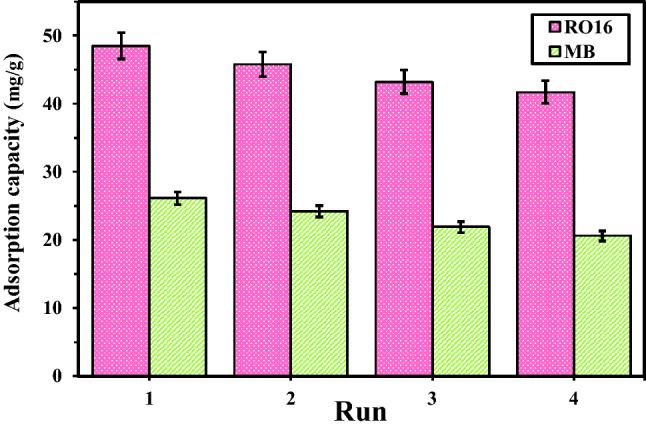
Reusability of MR-LDH for RO16 and MB adsorption.

Reusability is an economic factor in adsorption fields on laboratory and industrial scales. Since MR-LDH is a magnetic adsorbent, collecting it with an external magnet is convenient. After washing the collected adsorbent and reusing it four consecutive times, there is the ignorable decline in adsorption efficiency in the performance of MR-LDH, as displayed in Fig. 14. It shows that the collection and washing of the adsorbent can discharge the analyte from the interlayer of MR-LDH, leading to regenerating the sorption sites with high efficiency. Thus, we believe that our adsorbent shows acceptable reusability in repetitive cycles.

### Comparison with other adsorbents

Since it has been estimated that 12% of dyes are wasted during the production process, numerous adsorbents were presented to eliminate the dye molecules from environmental wastewater. Herein, we compare our proposal adsorbent, MR-Co/Al LDH, with previous reports to highlight the benefits of present works. All the caparisoned items are shown in Table 5. As can be seen, our material is superior compared to those reported earlier. In addition to the optimum adsorption conditions, our adsorbent takes advantage of the magnetic property. Therefore, an external magnetic field can easily separate them from the solution after the adsorption. Thus, it can be favorable for a reusability strategy.

**Table 5:** Comparison with other adsorbents for the removal of MB and RO16.

Adsorbent	Adsorbate	Adsorption capacity (mg/g)	References
g-C_3_N_4_@NiCo LDH	MB	25.06	^70^
Agadiite-chitosan composite beads	MB	45.25	^71^
Mesoporous silica nanoparticles	MB	19.26	^72^
Fe-BDC MOF	MB	8.65	^73^
Orange and lemon peels-derived activated carbon	MB	38	^74^
Silver nanoparticles	MB	218.95	^75^
MR-Co/Al LDH	MB	54.01	This work
Modified zeolites (cyclone ash)	RO16	12.6	^76^
Arachis hypogaea pod powder	RO16	56.48	^77^
Quartenised sugar cane bagasse	RO16	34.48	^78^
Activated carbon prepared from rice husk ash	RO16	13.32	^79^
AC@nZVI/Ni)	RO16	10.53	^80^
MR-Co/Al LDH	RO16	53.04	This work

## Conclusions

Summarily, a magnetic core–shell, namely MR-LDH, was synthesized as previously reported. Removal of organic dyes has become a worldwide challenge as a result of arising various human diseases. The as-prepared material was exposed to the removal of two organic dyes, MB and RO16, which had an adsorption capacity of 54.01 and 53.04 mg/g, respectively. The primary parameters affecting the adsorption process were optimized, including pH, contact duration, and adsorbent dose. Then, the modeling calculations were performed to provide a better understanding of adsorption mechanisms. Accordingly, the Langmuir isotherm was well fitted with both dyes adsorption experiments demonstrating the monolayer adsorption process. Additionally, the pseudo-second-order model provided the best model of the adsorption kinetics of both dyes. Lastly, reusability is essential for realizing efficient, economical, and environmentally friendly adsorbents. Since an external magnet can effortlessly collect the magnetic adsorbent, it provides a facile platform for eliminating dyes from wastewater which is required to expand appropriate cases in the social application.

## Data Availability

All data generated or analyzed data for experimental part during this study are included in this published article. The data that support the findings of this study are available from the corresponding author, [Asiyeh kheradmand], upon reasonable request. Moreover, all other data that support the plots within this paper and other findings of this study are available from the corresponding author upon reasonable request.
